# Starch Aldehyde–Theaflavin Conjugate: Synthesis, Structure, and Antioxidant and Antimicrobial Activities

**DOI:** 10.3390/foods15030487

**Published:** 2026-02-01

**Authors:** Yundong Shao, Yong Cheng, Xingqian Ye

**Affiliations:** 1 National-Local Joint Engineering Laboratory of Intelligent Food Technology and Equipment, College of Biosystems Engineering and Food Science, Zhejiang University, Hangzhou 310058, China; 2 Zhejiang Key Laboratory of Agri-Food Resources and High-Value Utilization, College of Biosystems Engineering and Food Science, Zhejiang University, Hangzhou 310058, China; 3 Zhejiang Skyherb Biotechnology Inc., Hangzhou 310015, China

**Keywords:** theaflavin, starch aldehyde, conjugate, antioxidant, antimicrobial activity

## Abstract

In the present study, potato starch (PS) was functionalized with theaflavin (TF). Potato starch aldehyde (DPS)–theaflavin (DPS-TF) conjugates were prepared by conjugating TF with DPS. The synthesized DPS-TF conjugates were characterized via UV–visible spectroscopy, Fourier transform infrared spectroscopy (FTIR), X-ray diffraction (XRD), proton nuclear magnetic resonance (^1^H-NMR) and scanning electron microscopy (SEM) analysis and tested for antioxidant and antimicrobial activities. The UV–vis spectrum results demonstrated that DPS-TF conjugates exhibited the characteristic absorption peaks of theaflavin at 280 nm, which can be attributed to the benzotropolone structure present in theaflavin. The absorbance values of the peaks progressively intensified as the concentration of grafted theaflavins increased. FTIR confirmed the depletion of the aldehyde groups and the presence of TF-specific vibrations in the conjugates in DPS-TF. ^1^H-NMR demonstrated that the conjugation occurred between the H-6, H-8, H-6′, and H-8′ positions of theaflavin and the aldehyde groups of starch aldehyde. XRD demonstrated that the DPS-TF conjugates were in the amorphous state. SEM observation demonstrated that DPS-TF exhibited a mixed morphology of flakes and lumps, which differed from that of native starch and starch aldehyde. The scavenging activity of DPS-TF against DPPH and ABTS radicals was significantly higher than that of DPS (*p* < 0.05), with the antioxidant activity increasing in line with the concentration of theaflavins. In comparison with PS and DPS, DPS-TF conjugates demonstrated superior antimicrobial activity against *Escherichia coli* and *Staphylococcus aureus*. Furthermore, an elevated grafting ratio corresponds to a heightened level of these functional properties. This study highlights the promise of the starch aldehyde–theaflavin conjugates for use as a viable antioxidant and antimicrobial agent for food applications.

## 1. Introduction

Starch, a plant-derived polysaccharide, is widely used in the food industry owing to its abundant availability, complete biodegradability, low cost, renewability, and non-toxic nature [1]. In the domain of food production, it fulfils a variety of functions, including those of a gelling agent, thickener, emulsion stabilizer and packaging material [2,3,4,5]. However, native starch’s inherent shortcomings, including water insolubility, brittleness, recrystallization tendency, high hydrophilicity and lack of inherent functional activity, severely restrict its application in the food sector [6,7,8]. To overcome these drawbacks, a multitude of modification techniques, including physical, chemical, and enzymatic methods, are employed [9,10,11,12]. Among these, chemical modification is the most widely used method. Oxidation is a pivotal chemical modification that selectively converts hydroxyl groups on the starch polymer chain into aldehyde or carboxyl groups [11,13]. The introduction of aldehyde groups, in particular, will enhance the performance of oxidized polysaccharides. Aldehyde groups provide starch with extra antibacterial and antioxidant properties that are crucial for food applications [14,15,16]. In addition, these carbonyl moieties are highly reactive, serving as key sites for further chemical conjugation through reactions such as Schiff base formation with primary amines, enabling the grafting of various bioactive molecules [11,17]. Potato starch, a carbohydrate extracted from the tubers of Solanum tuberosum, is a widely used renewable polymer in both food and non-food industries.

To date, several methods have been successfully applied to covalently link starch aldehyde and polyphenols, including chemical coupling, radical-mediated grafting, and acid-catalyzed condensation reactions [18,19,20]. Among these, acid-catalyzed condensation offers a relatively simple and safe route for synthesizing polysaccharide–polyphenol conjugates. This approach generally involves the introduction of aldehyde groups into the starch backbone, often through controlled oxidation, to create reactive sites. These reactive sites subsequently undergo covalent coupling with polyphenol molecules via acid-catalyzed reactions, including acetal formation or Schiff base reactions. This acid-catalyzed polycondensation is favored due to the relatively mild conditions and the formation of stable covalent bonds [18]. Using this acid-catalyzed condensation strategy, researchers have synthesized various conjugates, such as dialdehyde starch, with quercetin, catechin, epicatechin, epicatechin gallate, epigallocatechin, and epigallocatechin gallate. These conjugates have shown favorable grafting ratios and exhibited potent antioxidant activity [16,18,21]. Further evidence indicates that the antioxidant capacity of aldehyde-modified starch can be markedly improved after conjugation with antioxidant polyphenols, with the grafted polyphenolic moieties being the primary contributors to the observed antioxidant effects. Moreover, starch dialdehyde–polyphenol conjugates are non-toxic and demonstrate strong antimicrobial properties [12,16,22].

Theaflavin (TFs), water-soluble pigments formed during the processing of black tea, are primarily formed by the oxidation and condensation of catechins and their derivatives under the catalytic action of enzymes such as polyphenol oxidase (PPO) and peroxidase (POD), resulting in the formation of flavanol compounds [23]. The four primary theaflavins, including theaflavin (TF1), theaflavin-3-gallate (TF2a), theaflavin-3′-gallate (TF2b), and theaflavin-3,3′-digallate (TF3), are renowned for their exceptional biological properties [24]. TFs possess a wide range of biological activities, including antioxidation, antibacterial, alleviation of metabolic syndrome, anti-inflammation, neuroprotection, and anti-depression properties, and thus hold broad application prospects [25,26]. Theaflavin is a potent free radical scavenger that can directly eliminate reactive oxygen species (ROS) and inhibit their production, showcasing its potential as a potent natural antioxidant [27,28,29]. Studies have demonstrated the broad-spectrum antimicrobial activity of TFs, which effectively suppress pathogenic Gram-negative and Gram-positive bacteria and fungi [30,31,32]. Despite their significant advantages, TFs are chemically unstable; susceptible to degradation under light, heat, and alkaline conditions; and have limited bioavailability, which hinders their practical application [33]. To date, it remains unknown whether theaflavins can be successfully grafted onto polysaccharides and whether the resulting conjugates possess significant antioxidant and antimicrobial activities.

To the best of our knowledge, the conjugation of theaflavin onto starch aldehyde has not been investigated. This study was the first to efficiently conjugate theaflavin to potato starch using an acid-catalyzed condensation method. To synthesize the conjugate, starch was first subjected to oxidation using sodium periodate (NaIO_4_) to obtain aldehyde-functionalized starch. This intermediate was subsequently reacted with theaflavin under acid catalysis to form the starch-theaflavin conjugate. The successful graft was verified through comprehensive characterization using UV–visible spectroscopy, FTIR, SEM, XRD and ^1^H- NMR methods. The antioxidant and antimicrobial properties of the conjugate were also evaluated, and the results highlight its potential as a novel strategy for developing functional starch-based materials.

## 2. Materials and Methods

### 2.1. Materials and Chemical Reagents

Theaflavin (62.1% purity) was provided by Zhejiang Sky Herb Biotechnology Co., Ltd. (Hangzhou, China). Chemical reagents, including sodium periodate, dimethyl sulfoxide, sodium phosphate and Folin–Ciocalteu reagent, were sourced from Macklin Co. (Shanghai, China). Potato starch, hydrochloric acid (HCl, 36.0–38.0%) and sodium hydroxide (NaOH) were procured from Sinopharm Chemical Reagent Co. Ltd. (Shanghai, China). The bacterial strains (*Escherichia coli* and *Staphylococcus aureus*) were kindly supplied by the Food Microbial Laboratory of Zhejiang University (Hangzhou, China).

### 2.2. Preparation of Starch Aldehyde

Potato starch aldehyde was synthesized according to the procedure reported by Zuo et al. [34]. In brief, 6.5 g of sodium periodate was dissolved in 50 mL of distilled water, after which 10 g of potato starch was gradually introduced into the solution. The pH was adjusted to 3.5 using 1 mol/L hydrochloric acid aqueous solution. Oxidation was carried out in the dark at 35 °C for 4 h. The resulting products were subsequently washed ten times with distilled water, dialyzed against distilled water for 72 h, and finally freeze-dried to obtain the starch aldehyde. The yield of starch aldehyde was 43.2%.

### 2.3. Analysis of Aldehyde Groups in Starch Aldehyde

The aldehyde group content in starch aldehyde was quantified using a rapid alkali consumption method [35]. Briefly, 0.1 g of the sample was dissolved in 5 mL of 0.25 mol/L NaOH aqueous solution at 70 °C for 2 min and subsequently cooled to room temperature. To this solution, 7.5 mL of 0.125 mol/L sulfuric acid aqueous solution, 15 mL of distilled water, and 0.5 mL of 0.2% (*w*/*v*) neutral phenolphthalein ethanol solution were sequentially added. The resulting mixture was then titrated with 0.25 mol/L sodium hydroxide aqueous solution. The titration endpoint was identified by the appearance of a persistent purple coloration that remained stable for at least 30 s. Additionally, native starch samples were also titrated as a control. The percentage of dialdehyde units was given by the following equation:

aldehyde group content % = C1V1−2C2V2(W161)×1000×100

where C_1_ and C_2_ are the concentrations (mol/L) of NaOH and H_2_SO_4_ aqueous solution, respectively. V_1_ and V_2_ are the total volumes of NaOH and H_2_SO_4_ aqueous solutions used, respectively. W is the mass of the DPS sample, and 161 is the average molecular weight of the repeat unit in DPS. Each sample was repeated in triplicate.

### 2.4. Preparation of Starch Aldehyde–Theaflavin Conjugates

Following the method of Yong et al. [18], starch aldehyde–theaflavin conjugates were synthesized. Briefly, 0.1 g of starch aldehyde was dissolved in 3.3 mL of 1 mol/L HCl, while varying amounts of theaflavin (0.05, 0.1, and 0.15 g) were separately dissolved in 6.7 mL of DMSO. The two solutions were combined and allowed to react at 40 °C in the dark for 48 h. The product was then dialyzed against distilled water for 72 h. Based on the quantity of theaflavin added, the resulting conjugates were designated as starch aldehyde–theaflavin conjugate I (DPS-TF I), starch aldehyde–theaflavin conjugate II (DPS-TF II), and starch aldehyde–theaflavin conjugate III (DPS-TF III). All samples were stored at −20 °C in a desiccator until further analysis.

### 2.5. Grafting Ratio of Starch Aldehyde–Theaflavin Conjugates

The grafting ratio of the starch aldehyde–theaflavin conjugates was analyzed using the Folin–Ciocalteu method. In brief, 1 mL of an aqueous solution of the conjugate (0.1 mg/mL) was mixed with 1 mL of Folin–Ciocalteu reagent (10-fold diluted) and incubated at 30 °C for 5 min in the dark. After adding 5 mL of saturated sodium carbonate solution, the mixture was incubated for a further 2 h under the same conditions. The absorbance was measured at 760 nm. The grafting ratio was quantified based on a theaflavin standard curve (y = 6.9759x + 0.022) and expressed as mg theaflavin equivalents per gram of conjugate (mg TE/g).

### 2.6. Analysis of Aldehyde–Theaflavin Conjugates

#### 2.6.1. UV–Visible Spectroscopy

UV–vis spectrum was recorded by scanning sample aqueous solutions from 200 to 600 nm on a Lambda 35 spectrophotometer (PerkinElmer Ltd., Waltham, MA, USA) to distinguish whether the test sample contained grafted theaflavins, based on the characteristic UV absorption peak of theaflavins, which differs from that of starch aldehydes.

#### 2.6.2. Fourier Transform Infrared Spectroscopy (FTIR) Analysis

The sample was scanned using a Fourier transform infrared spectroscopy device (Cary 610/670, Varian, Palo Alto, CA, USA) within a wavenumber range of 4000–400 cm^−1^ at a resolution of 4 cm^−1^,

#### 2.6.3. X-Ray Diffraction (XRD)

X-ray diffraction patterns were analyzed using the method of Dong et al. [36]. The crystal structure was measured using a D8 Advance polycrystalline X-ray diffractometer (D8 Advance, Bruker AXS, Karlsruhe, Germany) at 40 kV and 40 mA with a scanning range (2θ) of 5–70°.

#### 2.6.4. Scanning Electron Microscopy (SEM)

The sample was evenly glued on the conductive tape of the sample table; then, more powder was blown through, and it was gold-sprayed, observed and photographed using GeminiSEM 300 equipment (Carl Zeiss, Oberkochen, Germany) at a voltage of 5 kV with a magnification of 1000×.

#### 2.6.5. Proton Nuclear Magnetic Resonance (^1^H-NMR) Spectroscopy

An Agilent DD2 spectrometer (Agilent Technologies Inc., Santa Clara, CA, USA) was used to record the ^1^H NMR spectra of the sample D2O solution at 25 °C and 400 MHz of operating frequency.

#### 2.6.6. Antioxidant Activity Assay

The antioxidant effect of DPS-TF conjugates was measured by means of scavenging 2, 2′-azino-bis-(3-ethylbenzothiazoline-6-sulphonic acid) diammonium salt (ABTS) and 2, 2-diphenyl-1-picrylhydrazyl (DPPH) radicals, with Trolox serving as a reference [37]. The scavenging activity of DPS-TF conjugates was expressed as μmol Trolox equivalent (TE)/g. For DPPH and ABTS radical scavenging assays, the specific wavelengths of the absorbance measurements were 517 nm and 734 nm, respectively.

#### 2.6.7. Antibacterial Activity Assay

*Escherichia coli* (*E. coli* ATCC 25922) and *Staphylococcus aureus* (*S. aureus* ATCC 29213) were used as experimental bacteria. The inhibitory effects of DPS-TF on bacteria were studied by the method of Yun et al. [38]. *E. coli* and *S. aureus* were cultured in LB agar liquid medium at 37 °C until reaching 10^8^ CFU/mL. The cultures were then diluted to 10^6^ CFU/mL with physiological saline and supplemented with 5 mg each of TF, PS, DPS, DPS-TF I, DPS-TF II, and DPS-TF III. Incubation continued at 37 °C for 24 h. Subsequently, the culture medium was diluted to a specific concentration and further incubated at 37 °C. After 24 h of incubation, the colonies were observed and counted. The antibacterial activity was expressed as the antimicrobial ratio (AR, %) and the logarithmic reduction in bacterial count. AR, % was calculated according to the following formula: AR (%) = (CFUcontrol − CFUsample)/CFUcontrol × 100%, where CFUcontrol and CFUsample are the colony-forming units per milliliter (CFU/mL) of the control and the sample-treated groups, respectively. Each sample was tested three times.

### 2.7. Statistical Analysis

The results were subjected to statistical analysis using the SPSS software, version 20. The data were expressed as the mean and standard deviation (SD). It was considered statistically significant when a *p*-value was less than 0.05.

## 3. Results and Discussion

### 3.1. Synthesis of Starch Aldehyde–Theaflavin Conjugates

**Figure 1:**
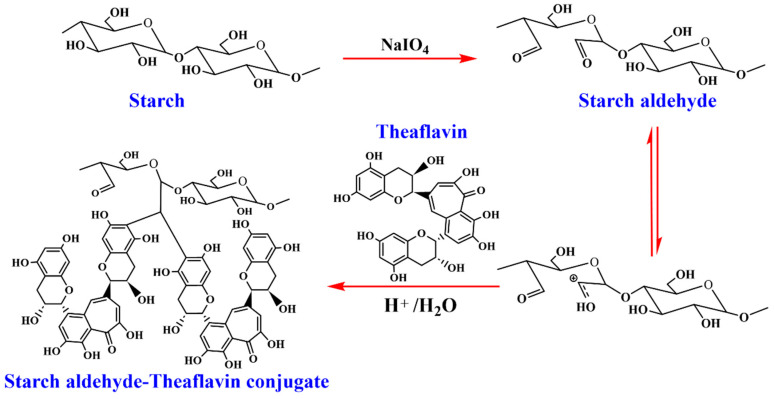
The proposed mechanism for the synthesis of starch aldehyde–theaflavin conjugate through acid-catalyzed condensation reaction.

Starch aldehyde is typically prepared by oxidizing native starch (from corn, potato, tapioca, etc.) with periodate salts, most commonly sodium periodate (NaIO_4_). This reaction specifically cleaves the C2–C3 bond of the anhydroglucose units, converting the vicinal diols into two aldehyde groups, forming dialdehyde starch (DAS) [39]. The degree of oxidation is a critical parameter, controlled by the molar ratio of periodate to anhydroglucose unit and the reaction time [40]. A higher degree of oxidation introduces more aldehyde sites for subsequent grafting but can also degrade the starch polymer chain, affecting its mechanical integrity [39]. The starch aldehyde–theaflavin conjugates were synthesized by acid-catalyzed condensation. As demonstrated in Figure 1, potato starch was oxidized with sodium periodate to produce starch aldehyde. The aldehyde content of the starch aldehyde was determined to be 101.3% using a rapid quantitative alkali consumption method. Aldehyde content is one of the key factors influencing the grafting rate of starch aldehyde with theaflavins. High aldehyde content is typically associated with excess sodium periodate and appropriate acidity [34]. In this study, the yield of starch aldehyde, expressed as a percentage of the mass of natural starch, was 43.2%, a result that is likely attributable to the concurrent oxidation by sodium periodate and acid hydrolysis during the preparation process [16]. The reaction of polysaccharides such as dextran, sodium alginate, and chitosan with sodium periodate leads to cleavage of their polymeric chains [41]. Among these, chitosan and alginates underwent more extensive depolymerization compared with dextran [16].

As illustrated in Figure 1, protonation of an aldehyde group in starch aldehyde generated a carbocation, followed by nucleophilic attack from two theaflavin molecules at their C–6 positions. This study prepared three types of starch aldehyde–theaflavin conjugates by adding varying amounts of theaflavin to the reaction system. The yields of DPS-TF I, DPS-TF II, and DPS-TF III were 10.4%, 62.4%, and 47.6%, respectively.

The grafting ratios of the three-starch aldehyde–theaflavin conjugates were 238.4, 303.4, and 382.3 mg TE/g, with the grafting ratio increasing as the amount of theaflavin in the reaction system increased. Yong et al. [18] reported a conjugation ratio of 87.3 mg QE/g for a starch aldehyde–quercetin conjugate synthesized via acid-catalyzed condensation. In a study by Hu et al. [16], three starch aldehyde–catechin conjugates prepared by acid-catalyzed condensation had conjugation ratios of 233.29, 270.17, and 341.31 mg CE/g. A positive correlation was observed between the conjugation ratio and the initial amount of catechin added. This is similar to our research findings.

### 3.2. UV–Vis Spectroscopy of Starch Aldehyde–Theaflavin Conjugates

**Figure 2:**
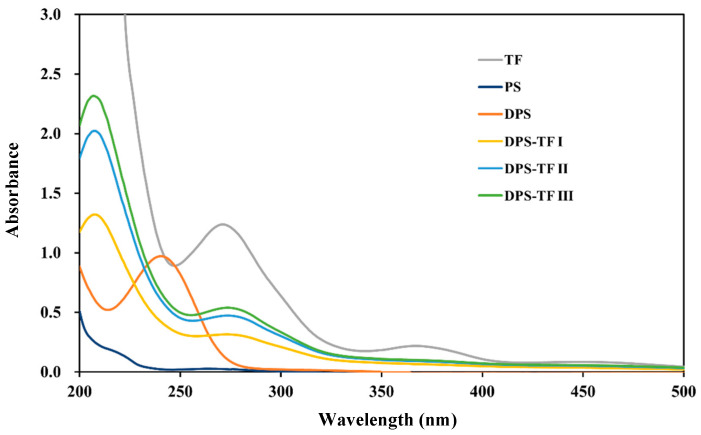
UV–vis spectra of TF, PS, DPS, DPS-TFI, DPS-TF II and DPS-TF III.

As shown in Figure 2, TF exhibited a maximum absorption peak for theaflavin at 280 nm, which is a characteristic absorption peak of the benzotropolone structure present in theaflavin. Another weaker absorption peak at 365 nm was observed in TF, which is another highly characteristic absorption peak of theaflavin. This peak originates from its extended conjugated system. The starch solution exhibits no absorption peaks in the UV–vis spectrum due to the absence of chromophores. DPS displayed a characteristic absorption peak at 240 nm corresponding to an aldehyde group [16,18]. However, the DPS-TF conjugate does not display this characteristic aldehyde absorption peak, indicating that the aldehyde group of DPS participated in the graft copolymerization reaction. In contrast, DPS-TF conjugates exhibited the characteristic absorption peaks of theaflavin at 280 nm, indicating successful grafting of theaflavin onto DPS. Furthermore, an increased conjugation ratio results in a higher absorption intensity in the DPS-TF conjugate. The UV–vis spectrum of the cassava starch aldehyde–catechin conjugate reported by Hu et al. [16] was also consistent with this finding.

### 3.3. FTIR Spectrum of Starch Aldehyde–Theaflavin Conjugates

**Figure 3:**
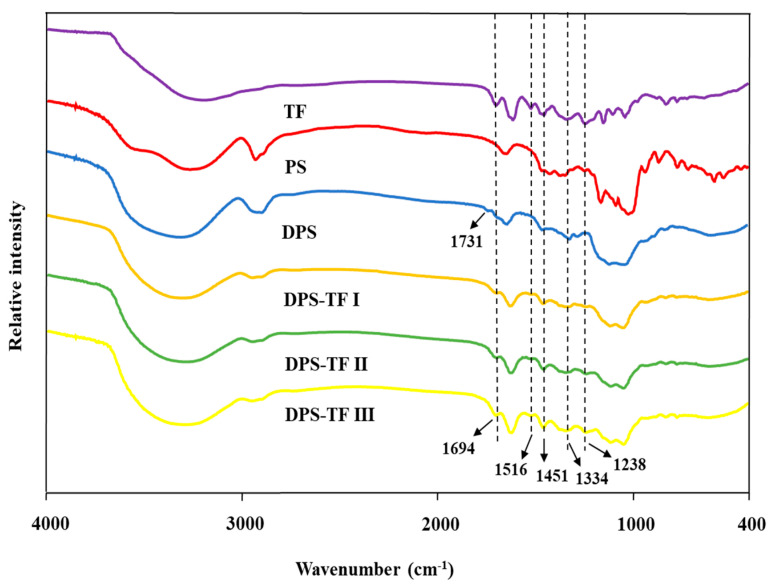
FT-IR spectra of TF, PS, DPS, DPS-TFI, DPS-TFII and DPS-TF III.

The FTIR spectra of theaflavin, starch, starch aldehyde, and starch aldehyde–theaflavin conjugate are shown in Figure 3. The FTIR spectrum of starch showed characteristic absorption bands at 3000–3600, 2800–3000, 1200–1500, and 900–1200 cm^−1^, which are attributed to O–H stretching, C–H stretching, C–O–H bending, and C–O–C stretching, respectively [39]. In contrast to native starch, DPS exhibited a novel band at 1731 cm^−1^, which was responsible for the carbonyl stretching vibration (C=O) of the aldehyde groups (–CHO) introduced during the periodate oxidation of starch [18,40]. The aldehyde group serves as the active site on DPS that reacts with TF. The peak at 1731 cm^−1^ in the DPS spectrum becomes nearly undetectable in the DPS-TF conjugates, directly demonstrating that the aldehyde group is extensively consumed during the grafting reaction and participates in the formation of covalent bonds. Two characteristic peaks at 1516 cm^−1^ (aromatic C=C stretching vibrations in TF’s benzotropolone core) and 1451 cm^−1^ (C–H in-plane bending vibrations of aromatic rings or -CH_2_- scissoring) appear in the DPS-TF spectra, matching those in pure TF but absent in DPS [41]. The presence of these TF-specific vibrations in the conjugates confirms successful incorporation of TF into the starch matrix. The maintained peak positions with slight broadening suggest that TF molecules experience a new chemical environment due to covalent attachment rather than physical confinement. The aforementioned findings suggest that theaflavin was effectively covalently conjugated onto DPS.

### 3.4. XRD Analysis of Starch Aldehyde–Theaflavin Conjugates

**Figure 4:**
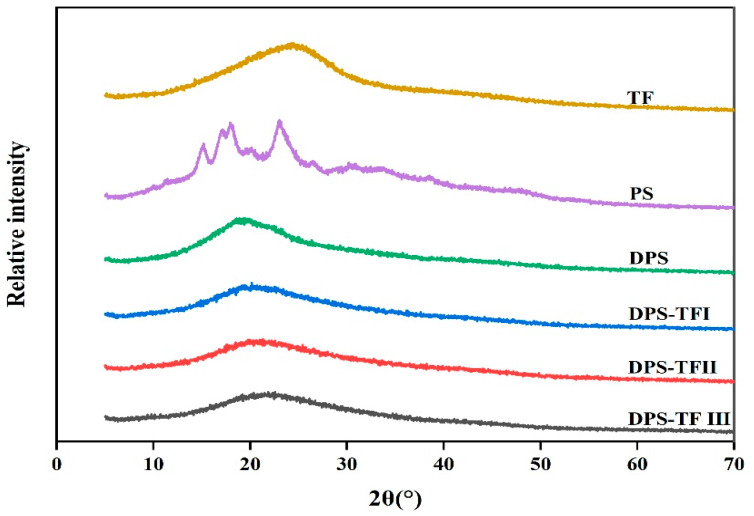
XRD pattern of TF, PS, DPS, DPS-TFI, DPS-TFII and DPS-TF III.

Figure 4 demonstrated the crystalline characteristics of theaflavin, starch, starch aldehyde and starch aldehyde–theaflavin conjugates. Theaflavins show a broad peak around 2θ = 24.6°, indicative of their amorphous nature. PS exhibits diffraction peaks at 2θ = 15.8°, 17.6°, 18.5° and 22.9°, indicating that the surface potato starch possesses a B-type semi-crystalline structure [42]. However, DPS exhibits a diffraction peak at 2θ = 19.9°, corresponding to its amorphous structure. The sodium periodate oxidation process significantly disrupts the original B-type crystalline structure of potato starch, transforming it from a semi-crystalline structure into a highly amorphous structure [18].

Three DPS-TFs conjugates exhibit an amorphous peak at 2θ = 23°, but the conjugate exhibited a much broader and weaker amorphous peak than the DPS, suggesting that its inter- and intra-molecular interactions were largely disrupted following conjugation with TFs [19]. This phenomenon can be attributed to the insertion of TF molecules into the structure of the starch chains, thereby disrupting the hydrogen bond network and crystal stacking. As the concentration of TFs increases, the intensity of the diffraction peaks decreases progressively, and the crystallinity further declines. The higher the grafting rate, the more pronounced the disruption to the crystalline structure. Consistent with our findings, other researchers have obtained analogous results with starch aldehyde–polyphenol conjugations [16,20].

### 3.5. ^1^H-NMR Spectra of Starch Aldehyde–Theaflavin Conjugates

**Figure 5:**
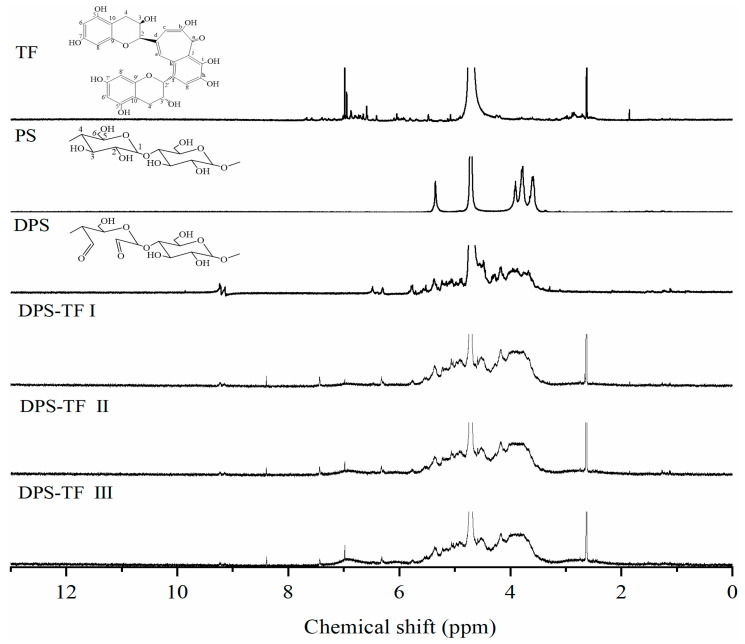
^1^H-NMR spectra of TF, PS, DPS, DPS-TFI, DPS-TFII and DPS-TF III.

Figure 5 demonstrates the ^1^H-NMR spectra of theaflavin, starch, starch aldehyde and starch aldehyde–theaflavin conjugates. Theaflavins showed phenylpyrone proton signals at δ = 7.67, 7.39, and 7.01 ppm, corresponding to H-g, H-e, and H-c, respectively. δ = 5.96, 5.92, 5.91, 5.80, 5.48, 5.08, 4.25, 4.19, 2.88, and 2.86 ppm correspond to H-6′, H-8′, H-6, H-8, H-2′, H-2, H-3′, H-3, H-4′, and H-4 on the o-cresol structure, respectively. For DPS-TF, the signal at δ = 7.01 ppm gradually intensified with an increasing grafting ratio (DPS-TFI to DPS-TFIII). However, no corresponding proton signals appeared at 5.80–5.96 ppm compared to the theaflavin, confirming graft copolymerization at theaflavin positions H-6, H-8, H-6′, and H-8′.

Starch showed a signal at δ = 5.35 ppm corresponding to the anomeric hydrogen proton on the glucose unit of starch, while the signals at δ = 3.91, 3.77, and 3.59 ppm correspond to the remaining hydrogen atoms (H2-H6) on the sugar ring of the glucose unit [43]. In comparison with native starch, starch aldehyde manifests more intricate proton signals at δ = 3.0–4.5 ppm. This phenomenon can be attributed to the ability of sodium periodate to disrupt the glucose ring structure, thereby creating a more complex chemical environment around the atoms. Furthermore, the characteristic signal of native starch (δ = 5.35 ppm) is found to be relatively weakened. It is noteworthy that DPS displayed two new proton signals at δ= 9.23 and 9.14 ppm, which are predominantly ascribed to the formation of the aldehyde group [16,44]. This signal progressively diminished in DPS-TF I/II and vanished in DPS-TF III, confirming aldehyde group participation in the grafting reaction.

All grafted products (DPS-TF I, II, III) exhibit a new signal at δ = 7.43 ppm, absent in both DPS and TF, primarily attributable to the shift in H-g and H-e. At δ = 7.01 ppm, the signal gradually intensifies with an increasing grafting ratio. However, in contrast to theaflavin, no corresponding proton signal was detected at 5.80–5.96 ppm, thereby confirming that graft copolymerization of theaflavin occurred at H-6, H-8, H-6′, and H-8′. The 1H NMR spectrum further provided evidence for the covalent grafting of TF onto DPS to form DPS-TF conjugates.

### 3.6. SEM Analysis of Starch Aldehyde–Theaflavin Conjugates

**Figure 6:**
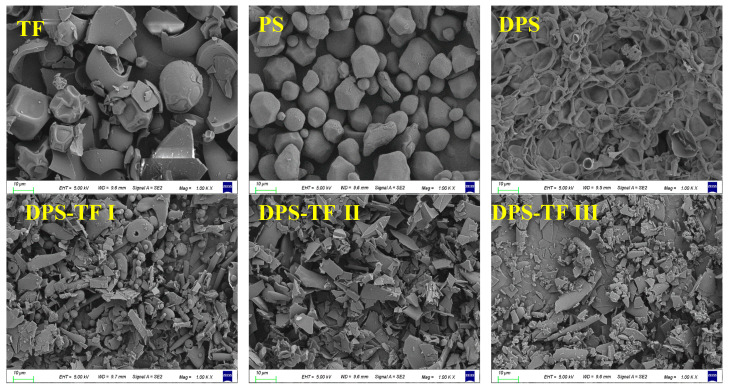
SEM images of TF, PS, DPS, DPS-TFI, DPS-TFII and DPS-TF III (magnification, 1000×).

The morphology of theaflavin, starch, starch aldehyde, and starch aldehyde–theaflavin conjugates was analyzed using SEM. As shown in Figure 6, theaflavin exhibits morphological irregularities, featuring a relatively dense surface structure accompanied by wrinkles and cracks resulting from shrinkage. Natural potato starch granules are predominantly distinct oval, ellipsoidal, or shell-shaped particles, with sizes ranging from 5 to 25 µm. It was observed that the smaller starch granules exhibited a spherical shape, the medium ones had an ellipsoid shape, and the large ones had an elongated, irregular shape. This finding aligns with previous reports concerning the morphology of starch granules [45,46].

After oxidation by sodium periodate on the potato starch surface, the morphology of starch particles is destroyed, resulting in a concave, wrinkled morphology. Following grafting with theaflavin, a significant change in the morphology of DPS was observed. The DPS-TF conjugate exhibited a mixed morphology of flakes and lumps. The structural transformation of the conjugates was evidenced by the absence of the crystalline structures of theaflavins and a loss of granular morphology, which is consistent with the XRD results that indicated a transition to an amorphous state. The disappearance of natural starch morphology and its encapsulation by an amorphous layer in DPS-TF samples confirms the successful grafting of theaflavins. In addition, the surface roughness of the DPS-TF conjugates increases with rising theaflavin concentration, indicating that the grafting rate enhances as the concentration increases.

### 3.7. Antioxidant Activity of Starch Aldehyde–Theaflavin Conjugates

**Figure 7:**
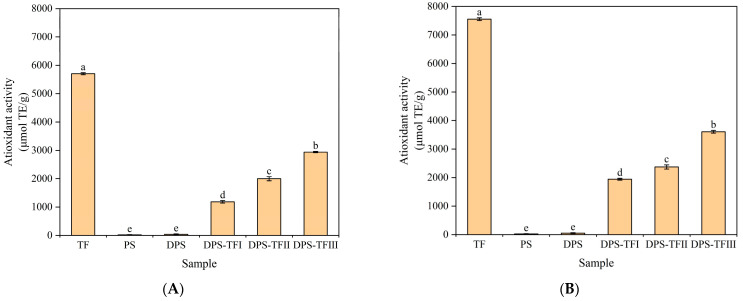
DPPH radical (**A**) and ABTS radical (**B**) scavenging activities of TF, PS, DPS, DPS-TFI, DPS-TFII and DPS-TF III. Each value represents mean ± SD of triplicates. Different lower-case letters in the same figure indicate a significant difference (*p* < 0.05).

The DPPH and ABTS radical scavenging ability of the compounds is a significant indicator of their antioxidant capacity. DPPH radical scavenging activity is a reliable indicator of an antioxidant’s capacity to eliminate free radicals by donating hydrogen atoms. ABTS radical scavenging activity is indicative of the ability of antioxidants to eliminate free radicals through electron transfer. As shown in Figure 7, theaflavin was found to demonstrate the most substantial antioxidant activity, as evidenced by the results of its DPPH and ABTS radical scavenging capacities. The phenolic hydroxyl group in the structure of theaflavin, particularly the pyrocatechol structure, is the key functional group responsible for its antioxidant activity [47]. The hydrogen atoms attached to this group are highly reactive and readily detached. Upon acquiring a hydrogen atom, the DPPH radical transforms into the stable non-radical compound DPPH-H. After losing its hydrogen atom, the theaflavin molecule forms a resonance-stabilized theaflavin oxygen radical. Due to the extensive conjugated system of its benzene ring structure, this radical possesses low energy and relative stability, preventing the initiation of new chain reactions and thereby terminating the radical damage process. The theaflavin directly donates an electron to ABTS. Upon receiving the electron, ABTS is reduced to the colorless neutral ABTS molecule. The theaflavin molecule itself, having lost an electron, is also oxidized to form the identical theaflavin oxygen radical [23].

As shown in Figure 7A,B, TF exhibited the greatest scavenging activity towards both the DPPH (5703.51 μmol TE/g) and ABTS (7554.48 μmol TE/g) radicals. PS and DPS exhibit very low DPPH and ABTS radical scavenging capacity. The radical scavenging activities (both DPPH and ABTS) of DPS-TF significantly surpassed those of its precursor, DPS, unequivocally confirming that the bioactive properties originate from the successfully grafted theaflavin moieties. After grafting theaflavin, the scavenging activity of DPS-TF increased with higher theaflavin concentrations. This is consistent with the results reported by Hu et al. [16], which indicated that the antioxidant activity significantly increased after the grafting of starch aldehyde and catechin and that the antioxidant activity was positively correlated with the amount of catechin grafted. The DPPH and ABTS radical scavenging capacity of DPS-TF is lower than that of free theaflavin, as the phenolic hydroxyl group involved in covalent bond formation cannot be reused for radical scavenging. Consequently, the final antioxidant activity of the grafted product is lower than that of the intrinsic polyphenol. A comparable tendency has been reported in relation to starch aldehyde–quercetin and starch aldehyde–catechin conjugates with regard to their DPPH and ABTS scavenging activity [22], which also suggests that the activity of free phenolic compounds was higher than that of the grafted material.

The scavenging activities at a specific concentration of DPS-TF against DPPH and ABTS radicals were more potent than those of dialdehyde guar gum–proanthocyanidins conjugates prepared under similar conditions [42]. The ABTS radical scavenging activity of starch aldehyde–theaflavin conjugate was found to be significantly higher than that of starch aldehyde–catechin conjugates and starch aldehyde–quercetin conjugates [16]. Theaflavins are dimeric oxidation products of catechins. Their distinctive benzoxepinone configuration demonstrates a superior capacity for free radical scavenging in comparison to the flavan-3-ol structure characteristic of catechins [43]. They contain a greater abundance of phenolic hydroxyl groups. This implies that even if a few hydroxyl groups are consumed during grafting, a substantial number of active hydroxyl groups remain on the molecule, thereby maintaining a high radical scavenging capacity [44]. This finding indicates that within the starch aldehyde–polyphenol copolymer system, the selection of a high-ranking antioxidant polyphenol (theaflavin) as the grafting ligand constitutes an effective strategy for improving its properties. In this study, the antioxidant activity determined by ABTS and DPPH exhibited similar behavior. Studies on diverse food extracts and polyphenol mixtures have repeatedly shown a high positive correlation between the outcomes of these two methods. In an analysis of 50 popular antioxidant-rich US foods, ABTS and DPPH assay results demonstrated a significant positive correlation [45], which is consistent with the results of this research. Both DPPH and ABTS assess antioxidant capacity through the single-electron transfer mechanism. The active phenolic hydroxyl group (-OH) in DPS-TF serves as the primary site for single-electron transfer reactions. Consequently, as the grafting ratio of grafted theaflavins increases, the total number of phenolic hydroxyl groups available to donate electrons in the system rises synchronously [45,46]. This leads to a similar, dose-dependent increase in scavenging rates measured by both methods. In this study, the starch aldehyde–theaflavin conjugate demonstrated superiority in antioxidant activity.

### 3.8. Antibacterial Activity of Starch Aldehyde–Theaflavin Conjugates

**Figure 8:**
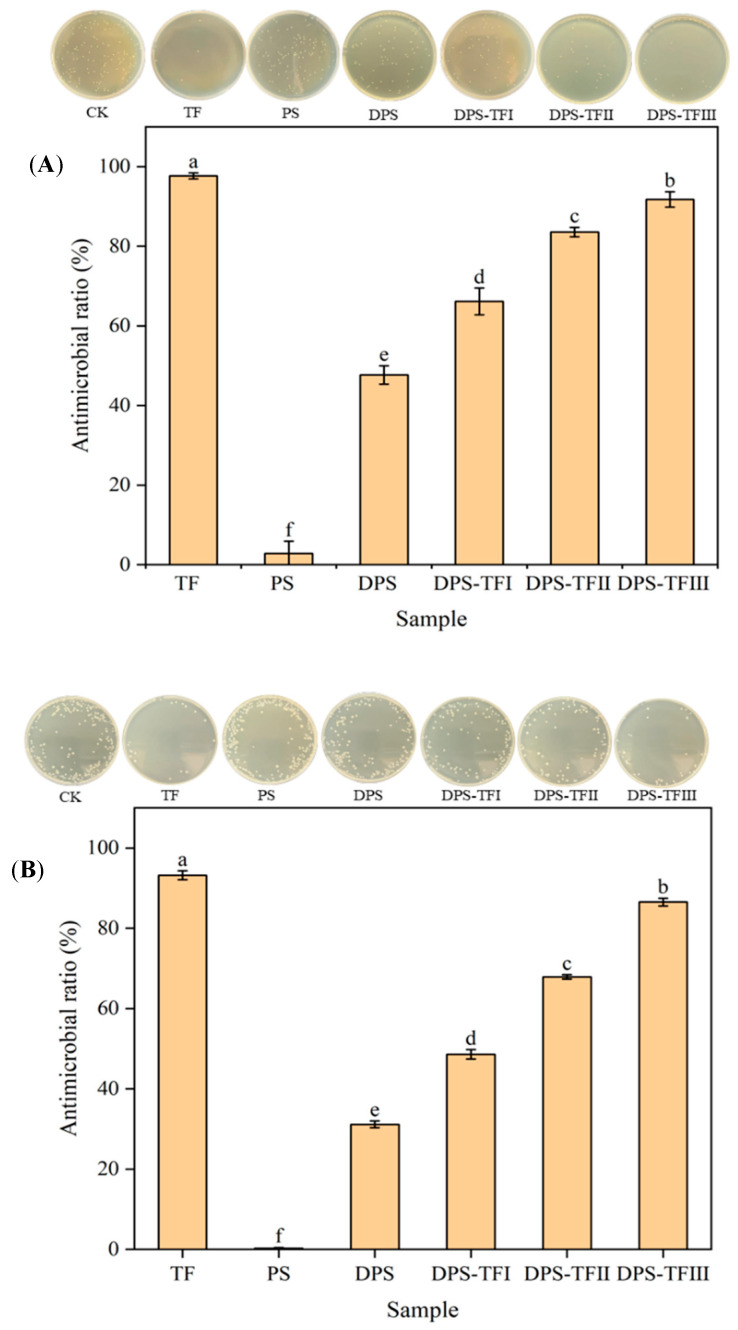
Antimicrobial ratio against *S. aureus* (**A**) and *E.coli* (**B**) of TF, PS, DPS, DPS-TFI, DPS-TFII and DPS-TF III. Each value represents mean ± SD of triplicates. The different superscript letters indicate a significant difference (*p* < 0.05).

To evaluate the antibacterial properties of the grafted copolymers, the antibacterial activities of the DPS-TF conjugates were evaluated against Gram-positive *S. aureus* and Gram-negative *E. coli*. As shown in Figure 8 and Table 1, theaflavins exhibited the strongest antibacterial activity. TF can interfere with and disrupt bacterial structure and function. TF can bind to bacterial surfaces to limit receptor attachment, disrupting bacterial cell walls and membranes to affect cell permeability, thereby inhibiting vital bacterial activities or inducing cell death [47,48,49]. Studies suggest that the hydroxyl group on TF’s benzophenone ring is pivotal to its antibacterial action, potentially through a mechanism akin to that of catechins. This mechanism involves TF–bacterial membrane interactions that inflict irreversible damage and alter key characteristics of pathogenic bacterial membranes [50].

Potato starch did not display any antimicrobial activity. However, the starch aldehyde form was much more effective in antibacterial properties. The observed antimicrobial activity of starch aldehyde was significantly enhanced when compared with native starch, which was primarily due to the presence of aldehyde groups. The study by Song et al. also revealed the strong antimicrobial effects of aqueous starch aldehyde solutions against *E. coli* and *S. aureus* [51]. The aldehyde group of starch aldehyde exerts its antimicrobial effect by directly forming irreversible cross-links with peptidoglycan and proteins in bacterial cell walls/membranes, rapidly disrupting bacterial structural integrity and altering permeability [15].

It is noteworthy that the antibacterial activity of starch aldehyde was further enhanced after it was grafted with theaflavin, and the higher the grafting rate, the stronger the antibacterial activity, which can be attributed to theaflavin’s potent antimicrobial activity [22,50]. Sun et al. [22] reported that the inhibitory activity of starch aldehyde–quercetin conjugate against *S. aureus* was positively correlated with its quercetin content, which is similar to our findings.

Although the absolute mass of TF in DPS-TF conjugate is lower than that in pure TF, the conjugate exhibited superior antibacterial activity. Research on the antibacterial activity of starch aldehyde–quercetin conjugate has revealed that, although quercetin exhibits the highest antibacterial activity, the graft conjugate also demonstrates remarkably high antibacterial activity [18,22]. Sun et al. [42] also found that equal amounts of dialdehyde guar gum–proanthocyanidin conjugates and proanthocyanidins exhibit comparable antibacterial activity. These results are consistent with the findings of this paper. The observation of comparable antibacterial effects specifically indicates a significant synergistic effect. On the one hand, this may be because the stability of TF was improved by conjugating it with DPS, which resulted in the exertion of sustained and stable antibacterial activity [52]. On the other hand, the comparable activity from a lower dose of the active ingredient demonstrates that the DPS-TF conjugate is a functionally integrated system. It delivers a high local efficacy for TF, making its antibacterial action far more efficient on a per-mass basis than the free compound. This is a significant advantage for potential applications, where it is crucial to minimize the amount of expensive bioactive compounds while maintaining high efficacy.

## 4. Conclusions

This study successfully established an acid-catalyzed condensation strategy for the precise synthesis of a starch aldehyde–theaflavin conjugate (DPS-TF). The covalent grafting of theaflavin onto the starch aldehyde backbone was unequivocally confirmed through a complementary suite of characterization techniques. UV–vis spectroscopy indicated the incorporation of theaflavin, while FTIR and ^1^H-NMR analyses provided direct evidence for the chemical conjugation. This molecular-level modification induced significant structural changes, as reflected by the increased amorphous characteristics revealed by XRD and the altered surface morphology observed via SEM, compared to both native potato starch and its aldehyde derivative. DPS-TF exhibited significantly stronger radical scavenging capacities (DPPH and ABTS) than its precursors. Its performance was superior to that reported for analogous conjugates with catechin and quercetin, underscoring the advantage of grafting the high-potency theaflavin molecule. In addition, the conjugate displayed potent antibacterial efficacy against both Gram-positive (*S. aureus*) and Gram-negative (*E. coli*) bacteria. Consequently, DPS-TF conjugates hold outstanding promise as a bioactive ingredient, particularly for food industry applications aimed at simultaneously preventing oxidative and microbial spoilage. Future research will focus on in vivo efficacy assessments and developing practical formulations for its industrial application in food systems.

## Figures and Tables

**Table 1:** Antimicrobial effect against *S. aureus* and *E.coli* of TF, PS, DPS, DPS-TFI, DPS-TFII and DPS-TF III. Each value represents mean ± SD of triplicates. The different superscript letters indicate a significant difference (*p* < 0.05).

Sample		Bacterial Concentration (log_10_ CFU/mL)	Log Reduction	Antimicrobial Ratio (%)
*S. aureus*	Control	3.11 ± 0.02 ^a^	-	-
	TF	1.46 ± 0.15 ^f^	1.65 ± 0.15 ^a^	97.69 ± 0.77 ^a^
	PS	3.10 ± 0.01 ^a^	0.01 ± 0.01 ^f^	3.08 ± 3.08 ^f^
	DPS	2.83 ± 0.02 ^b^	0.28 ± 0.02 ^e^	47.69 ± 2.31 ^e^
	DPS-TF I DPS-TF II DPS-TF III	2.64 ± 0.04 ^c^ 2.33 ± 0.03 ^d^ 2.02 ± 0.11 ^e^	0.47 ± 0.04 ^d^ 0.79 ± 0.03 ^c^ 1.09 ± 0.11 ^b^	66.15 ± 3.35 ^d^ 83.59 ± 1.18 ^c^ 91.79 ± 1.94 ^b^
*E. coli*	Control	3.44 ± 0.01 ^a^	-	-
	TF	2.27 ± 0.07 ^f^	1.17 ± 0.07 ^a^	93.21 ± 1.11 ^a^
	PS	3.44 ± 0.01 ^a^	0.00 ± 0.00 ^f^	0.24 ± 0.21 ^f^
	DPS	3.28 ± 0.01 ^b^	0.16 ± 0.01 ^e^	31.15 ± 0.84 ^e^
	DPS-TF I DPS-TF II DPS-TF III	3.15 ± 0.01 ^c^ 2.95 ± 0.01 ^d^ 2.57 ± 0.03 ^e^	0.29 ± 0.01 ^d^ 0.49 ± 0.01 ^c^ 0.87 ± 0.03 ^b^	48.61 ± 1.17 ^d^ 67.88 ± 0.56 ^c^ 86.55 ± 0.96 ^b^

## Data Availability

The original contributions presented in this study are included in the article. Further inquiries can be directed to the corresponding author.
